# Effects of Lung Expansion Therapy on Lung Function in Patients with Prolonged Mechanical Ventilation

**DOI:** 10.1155/2016/5624315

**Published:** 2016-03-29

**Authors:** Yen-Huey Chen, Ming-Chu Yeh, Han-Chung Hu, Chung-Shu Lee, Li-Fu Li, Ning-Hung Chen, Chung-Chi Huang, Kuo-Chin Kao

**Affiliations:** ^1^Department of Respiratory Care, Chang Gung University College of Medicine, 259 Wenhua 1st Road, Guishan District, Taoyuan City 33302, Taiwan; ^2^Department of Respiratory Therapy, Chang Gung Memorial Hospital, Chang Gung University College of Medicine, 5 Fu-Shing Street, Guishan District, Taoyuan City 33305, Taiwan; ^3^Department of Thoracic Medicine, Chang Gung Memorial Hospital, Chang Gung University College of Medicine, 5 Fu-Shing Street, Guishan District, Taoyuan City 33305, Taiwan

## Abstract

Common complications in PMV include changes in the airway clearance mechanism, pulmonary function, and respiratory muscle strength, as well as chest radiological changes such as atelectasis. Lung expansion therapy which includes IPPB and PEEP prevents and treats pulmonary atelectasis and improves lung compliance. Our study presented that patients with PMV have improvements in lung volume and oxygenation after receiving IPPB therapy. The combination of IPPB and PEEP therapy also results in increase in respiratory muscle strength. The application of IPPB facilitates the homogeneous gas distribution in the lung and results in recruitment of collapsed alveoli. PEEP therapy may reduce risk of respiratory muscle fatigue by preventing premature airway collapse during expiration. The physiologic effects of IPPB and PEEP may result in enhancement of pulmonary function and thus increase the possibility of successful weaning from mechanical ventilator during weaning process. For patients with PMV who were under the risk of atelectasis, the application of IPPB may be considered as a supplement therapy for the enhancement of weaning outcome during their stay in the hospital.

## 1. Introduction

As medicine and technology advance, increasing numbers of patients survive critical illnesses but experience difficulty being weaned off from their mechanical ventilator. The American Health Care Financing Administration defines prolonged mechanical ventilation (PMV) as requiring at least 6 h of mechanical ventilation per day for more than 21 consecutive days [1]. It has been estimated that 600,000 patients per year will be receiving PMV at a hospital cost of about 50 billion dollars in 2020 in the US [2]. Increasing the weaning rate and improving hospitalization outcomes for patients on PMV have become important issues [3].

Common complications in PMV include changes in the airway clearance mechanism, pulmonary function, and respiratory muscle strength, as well as chest radiological changes such as atelectasis [4, 5]. The underlying disease and presence of artificial airway result in excessive secretion, leading to airway obstruction and poor oxygenation [6]. PMV also impairs respiratory muscle strength, reduces lung volume, and affects the ability to perform deep breathing. Studies have shown patients with inadequate respiratory muscle strength to be prone to atelectasis, which often results in reduced lung volume and impairment of gas exchange [7, 8]. These conditions have been indicated as major reasons for extended mechanical ventilator duration and failure to be weaned from mechanical ventilation [9]. Interventions that facilitate lung expansion may prevent deterioration in pulmonary function and increase the weaning rate for patients on PMV [10].

Intermittent positive pressure breathing (IPPB) is a common therapy for lung expansion, delivering an inspiratory positive pressure for creating the pressure gradient required to cause airflow into the lungs [11]. In 1972, using IPPB to induce lung hyperinflation was reported to increase lung dynamic compliance by up to 70% in patients with kyphoscoliosis [12]. Recently, IPPB was shown to improve vital capacity in patients with neuromuscular diseases [13]. IPPB prevents and treats pulmonary atelectasis, helps mobilize secretion, and improves lung compliance in patients who have or are under the risk of pulmonary complication. In addition to IPPB, it has been proposed that positive end-expiratory pressure (PEEP) can promote a more homogeneous distribution of pulmonary ventilation through interbronchial collateral channels and prevent airway collapse during the expiratory phase [14]. These two benefits are essential for patients on PMV to regain normal pulmonary function. Whether the combination of IPPB and PEEP therapy produces better outcomes remains unclear.

Although the benefits of IPPB and PEEP therapy in pulmonary function have been established, few studies have investigated their effects in patients on PMV. The primary purpose of this study was to compare the short-term effects on pulmonary function of IPPB alone versus IPPB combined with PEEP therapy in patients on PMV. Our secondary purpose was to examine the effects of IPPB intervention on hospitalization outcomes of patients on PMV.

## 2. Methods 

### 2.1. Patients

This study was performed in the respiratory care center (RCC) of Chang Gung Memorial Hospital (CGMH), Taiwan. It was approved by the Institutional Review Board of CGMH, and the Informed Consent Form was obtained from the patients or their relatives before inclusion. The inclusion criteria were as follows: (1) mechanical ventilation for more than 6 h/day for more than 21 days and failure to be weaned from mechanical ventilation in ICUs; (2) medical stability (arterial blood gas value pH: 7.35–7.45, PaO_2_: 60 mmHg at 40% FiO_2_, absence of signs and symptoms of infection, and hemodynamic stability); (3) mechanical ventilation by pressure support with a mechanical ventilation weaning program scheduled; and (4) reduced lung volume (vital capacity <10 mL/kg or tidal volume (*V*_*t*_) <5 mL/kg). Exclusion criteria included cancer, acute lung or systemic infection, hemodynamic instability, active hemoptysis, tracheoesophageal fistula, or intracranial pressure >15 mmHg.

### 2.2. Study Protocol

This study was prospective and randomized. Randomization involved using sequential sealed envelopes prepared by an independent investigator before patient recruitment. Patients were assigned to IPPB, IPPB combined with PEEP (IPPB + PEEP), or control group according to the labels on envelopes randomly chosen by another investigator. The age, sex, body weight, height, and diagnosis at RCC admission of each patient were recorded, and the severity of disease was assessed with the Acute Physiology and Chronic Health Evaluation II score within 24 h of RCC admission.

In the IPPB or IPPB + PEEP group, patients received the intervention therapy in semi-Fowler's position. The IPPB treatment consisted of 20 min of IPPB twice a day for 7 days, provided by Bird Mark 7 (Viasys, USA). Inspiratory pressure was gradually increased to achieve the target volume of 10–15 mL/kg of predicted body weight. The inspiratory trigger and flow and respiratory rate were adjusted to maximize patient comfort. Similar devices and adjustment of inspiratory pressure were used for the IPPB + PEEP group, with an external PEEP valve attached to the expiratory port of the IPPB circuit; the PEEP level was adjusted according to the patient's previous ventilator PEEP setting. During each therapy session, patients were continuously monitored, with safety alarms for heart rate, respiratory rate (RR), blood pressure, electrocardiogram, and oxygen saturation by peripheral oximetry. Patients in the control group received the same medical treatment as those in the IPPB group, except for IPPB therapy. The weaning profile and arterial blood gas (ABG) data were measured on the day before intervention (day 0) and a day after completing the intervention (day 8). Research personnel recorded the lung mechanics data (lung compliance and airway resistance) from the mechanical ventilator in every therapy session.

### 2.3. Outcome Measures

Patients were required to maintain semi-Fowler's position during the measurement of weaning profiles. Minute volume (MV), *V*_*t*_, respiratory muscle strength (maximal inspiratory pressure, MIP), and RR were measured with an electrical mechanical monitor (Respiradyne II, Sherwood, USA). The rapid shallow breathing index, calculated as RR divided by *V*_*t*_, was recorded for the pulmonary function and weaning procedure. During the first and last therapy sessions, the total expectorated secretion was collected by suction and the wet sputum weight recorded. Resting ABGs were systematically analyzed (Corning 248 blood gas analyzer, Siemens RAPID Lab, USA) on days 0 and 8 in three groups. PaCO_2_, PaO_2_, pH, and PaO_2_/FiO_2_ were recorded.

The hospitalization outcomes were followed until patients were discharged from RCC. Survival status, weaning outcome, the duration of mechanical ventilation in RCC, and RCC length of stay were recorded from medical records. Weaning was considered successful if the patient was continuously free from the mechanical ventilator for more than 7 consecutive days.

### 2.4. Statistics Analysis

Data analysis was performed with SPSS software (version 17.0). Descriptive data was presented as means ± standard deviation. Paired *t*-tests were used to examine the effects of interventions on weaning profiles and lung mechanics within groups. Differences among the three groups in lung mechanics, weaning profiles, days of ventilator use, and the length of RCC stay were compared by one-way ANOVA. A Kruskal-Wallis test was used to analyze the differences of frequency distributions of mechanical ventilator weaning and survival rates between the groups. A *p* value <0.05 indicated statistical significance.

## 3. Results

**Figure 1:**
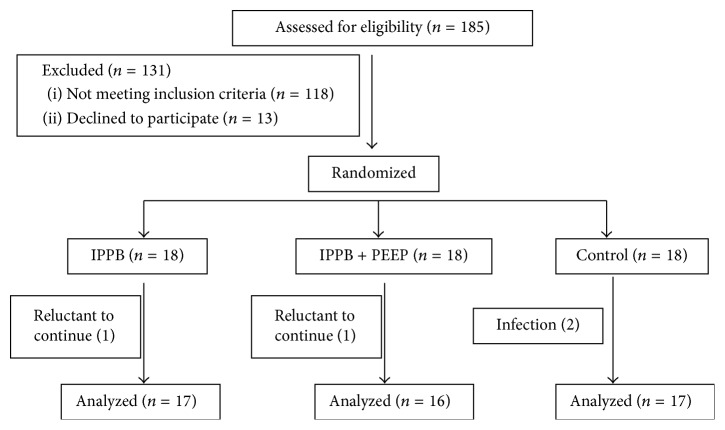
Flow chart of subjects participation and analysis.

Of 185 patients screened for the study between January 2012 and June 2013, 54 patients met the inclusion criteria. Two patients (one each from the IPPB and IPPB + PEEP groups) were reluctant to continue and ceased participation, and two control group patients were excluded due to episodes of acute infection (Figure 1). Fifty patients completed the program; their demographic and clinical characteristics are presented in Table 1. The three groups were similar with regard to age, body mass index, and disease severity. The mean ages in the IPPB, IPPB + PEEP, and control groups were 69.4, 76.4, and 72.3 years, respectively. Major diagnoses on admission to RCC were diseases that involved the pulmonary system.

Table 2 shows the weaning parameters and lung mechanics at the beginning and end of the intervention period. No differences were found in pre- and postmeasurements in the intergroup comparison. However, patients in the IPPB group showed a significant increase in *V*_*t*_ after the 7-day period (pre versus post: 240.4 ± 57.2 versus 292.5 ± 116.3 mL, *p* < 0.05), while the control group showed a significant reduction (pre versus post: 293.3 ± 168.9 mL versus 243.9 ± 140.4 mL, *p* < 0.05). In the IPPB + PEEP group, a significant increase in MIP was observed after the intervention period (29.9 ± 15.0 versus 37.0 ± 16.5 cmH_2_O, *p* < 0.05). Although there was a tendency for an improvement of lung compliance in the IPPB (48.2 ± 22.7 versus 55.4 ± 37.2 mL/cmH_2_O) and IPPB + PEEP groups (40.9 ± 22.4 versus 44.8 ± 24.8 mL/cmH_2_O), this was not statistically significant.

No significant differences in ABG were found in the pre- and postmeasurements between the groups. However, patients in the IPPB group had a significantly higher PaO_2_ (98.1 ± 21.9 mmHg versus 122.1 ± 32.4 mmHg, *p* < 0.05) and PaO_2_/FiO_2_ ratio (275.6 ± 64.5 versus 381.2 ± 112.2, *p* < 0.05) after the intervention (Table 3). The IPPB + PEEP group showed a significant decrease in pH (7.47 ± 0.03 versus 7.42 ± 0.05, *p* < 0.05). Control group patients showed no significant changes in ABG data. During the first session, patients in the IPPB + PEEP group had significantly more sputum than those in the IPPB and control groups (IPPB versus IPPB + PEEP versus control: 1.5 ± 1.4 versus 3.8 ± 2.3 versus 2.3 ± 1.3 g, *p* < 0.05). However, no significant difference between the groups was observed at the end of the intervention.

There was no significant difference in hospital survival rates between groups. As presented in Table 4, both IPPB and IPPB + PEEP groups had a significantly higher weaning rate than the control group (IPPB versus IPPB + PEEP versus control: 88.2% versus 87.5% versus 41.2%, *p* < 0.05). Although a shorter duration of RCC stay and lower mortality were observed in the IPPB group, the differences with the control group did not reach statistical difference. However, compared with the intervention groups, patients in the control group stayed longer on the mechanical ventilator during their stay in RCC (IPPB versus IPPB + PEEP versus control: 11.7 ± 3.7 days versus 15.8 ± 9.1 days versus 27.2 ± 16.1 days, *p* < 0.05).

## 4. Discussion

This study determined the effects of IPPB in patients on PMV by assessing changes in lung function and mechanics. We found that, after IPPB therapy, patients on PMV had significantly increased *V*_*t*_ and PaO_2_, whereas no significant improvements in lung volume were found in the control group. Patients in the IPPB + PEEP group demonstrated a significant increase in inspiratory muscle strength after intervention. Both intervention groups exhibited a higher successful ventilator weaning rate and fewer days on the mechanical ventilator than the control group.

In our study, 7-day IPPB therapy resulted in a significant improvement in *V*_*t*_ in patients on PMV. During the IPPB therapy, the application of positive pressure to the airway results in the airway opening, facilitating the distribution of inspired gas in the lungs. The increased recruitment of collapsed alveoli may lead to further improvement of lung volume. Guérin et al. examined the effects of IPPB on patients with neuromuscular diseases and reported a significant improvement in *V*_*t*_ in patients who received IPPB therapy [15]. However, Laffont et al. reported no change in vital capacity in patients with spinal cord injury after IPPB intervention [16]. This study differed from previous studies in both the diagnoses of the patients and the patients' baseline *V*_*t*_. For the IPPB group in our study, this was about 240 mL, similar to that in Guérin et al.'s study (250 mL) and approximately half the value of *V*_*t*_ of healthy adults [11]. In contrast, the mean *V*_*t*_ of patients in Laffont et al.'s study was about 634 mL, similar to the normal value of healthy adults. IPPB therapy is a lung hyperinflation therapy and is often indicated for patients with reduced lung volumes [17]. The results of our study raise the possibility that IPPB therapy may be beneficial for patients on PMV with reduced lung volumes to improve lung expansion.

The primary diagnosis may also have affected the therapy outcome. In our study, 41.2% of the patients in the IPPB group had primary problems that involved the neurological system on admission to RCC compared with only 18.6% of patients in the IPPB + PEEP group. As neuromuscular disease often contributes to restrictive pulmonary problems, one of the indications for IPPB therapy, the IPPB + PEEP group may have shown less improvement in *V*_*t*_ than the IPPB group because of the lower prevalence of neuromuscular diseases. However, compared with the control group, which showed a reduction in *V*_*t*_, IPPB + PEEP therapy may prevent the complications of being bedridden for a prolonged period in patients on PMV.

Our study showed that PaO_2_ and the PaO_2_/FiO_2_ ratio in the IPPB group had significantly increased at the end of the intervention period. Romanini et al. reported a significant increase in SpO_2_ after IPPB therapy in patients who underwent myocardial revascularization surgery [18]. Our results are consistent with the results of previous study [18] and indicate that IPPB is beneficial for improving oxygenation in patients prone to or already having hypoxemia. In conjunction with the increased *V*_*t*_ seen in our study, IPPB results in passive lung expansion by augmenting lung volume. Enhanced ventilation volume improves the ventilation/perfusion ratio and results in improved oxygenation status by increasing the efficiency of gas exchange. Improved oxygenation status is critical for patients on PMV, particularly when they are weaning themselves from the ventilator. During the weaning process, patients start to breathe without ventilatory assistance, and their oxygen consumption rises due to the increased work of breathing [19]. Patients are more likely to be successfully weaned from the mechanical ventilator when their capability for oxygen supply can overcome the added oxygen demand from the work of breathing. Improved oxygenation in the IPPB group may explain the higher weaning rate and shorter duration of mechanical ventilation compared with those in the control group. The reduction in days on the ventilator lowers complication risks such as ventilator-associated pneumonia and reduces health care costs and may improve the quality of life for the patients [20].

We observed a significant reduction in *V*_*t*_ in the control group. In patients on PMV, immobility and catabolism from the underlying illness and dependency on the ventilator are commonly present and often result in complications such as weakness of respiratory muscles, reduced lung volumes, or atelectasis [21]. The reduction of lung volumes may be caused by the cephalic shift of the diaphragm in the supine position combined with the gravitational load on the heart [22]. The abnormal diaphragm position may also result in an increase in nonaerated or underaerated lung regions. Although patients in all three groups remained on bed rest for most of the time in RCC, patients in the control group not receiving lung expansion therapy were thus at higher risk of deconditioning from prolonged bed rest.

Inspiratory muscle strength, as indicated by MIP, increased after patients completed IPPB + PEEP therapy. A previous study found that patients who underwent cardiac surgery had a significantly higher MIP than patients in the control groups after PEEP therapy. The physiological effects of PEEP increase functional residual capacity and reduce the work of breathing by keeping the alveoli and airways open during the expiratory phase [23]. The decrease in work of breathing may reduce oxygen consumption and the energy demand of respiratory muscles, thereby reducing muscle fatigue [24]. However, in our study, patients in the IPPB + PEEP group had a higher rapid shallow breathing index and lower MIP than other groups prior to the intervention, indicating a higher workload of breathing before participating in the study. The increase in MIP in this group may be due to the spontaneous recovery to baseline. As mentioned above, combined IPPB and PEEP therapy reduces the load on respiratory muscles and reduces muscle fatigue. The changes of MIP in IPPB + PEEP group (23.7%) were higher than in control group (6.8%) during 7-day periods. Given this, it is possible that applying IPPB + PEEP may accelerate the spontaneous recovery in inspiratory muscle strength over time. Further study is required to determine the possible effects of PEEP on respiratory muscle function.

Some limitations of this study should be considered when interpreting the results. First, although the number of patients in this study was comparable with that of previous studies that yielded positive results [15, 16], the small sample size and variation of diagnoses between groups may hamper the interpretation of results. Second, all participants received the same period of therapy to rule out any possible interference of variable sessions on the intervention effects. However, the positive effects of therapy on pulmonary function may be attenuated over time after discontinuing therapy. The beneficial effects of IPPB therapy on length of hospitalization may therefore be underestimated. Third, the expectoration of airway secretions was assessed only by measuring wet sputum weight, which may be influenced by factors such as the cause of infection and source of sputum [25]. The measurement of dried sputum weight may be more objective and could provide more information about the effects of therapy. Fourth, a previous study has reported that IPPB therapy reduces the risk of pulmonary complications during hospitalization [26]. This was not assessed in the present study. However, the significantly shorter number of days of mechanical ventilator use in the IPPB group may support the possibility that IPPB plays a role in preventing or reversing pulmonary complications in patients on PMV.

## 5. Conclusion

The use of IPPB therapy is beneficial for patients requiring prolonged mechanical ventilation in improving lung volume and oxygenation. IPPB therapy also reduces the number of days on the ventilator and increases the rate of successful weaning from the ventilator during the patients' stay in RCC. For patients requiring PMV, IPPB therapy may be considered to improve their pulmonary function and hospitalization outcomes.

## Figures and Tables

**Table 1:** Baseline characteristics of study participants.

	IPPB (*n* = 17)	IPPB + PEEP (*n* = 16)	Control (*n* = 17)
Age (yr)	69.1 ± 11.1	76.4 ± 14.7	72.3 ± 16.2
Sex (F/M)	10/7	8/8	5/12
APACHE II score	20.4 ± 3.9	22.0 ± 6.5	19.0 ± 5.8
Weight (kg)	56.9 ± 12.0	57.3 ± 14.5	59.4 ± 12.0
Height (cm)	159.5 ± 8.5	158.1 ± 8.7	161.4 ± 7.8
Ventilator status prior to study			
Ventilator days prior to study (days)	38.7 ± 20.2	36.9 ± 18.6	40.4 ± 21.6
PS level (cmH_2_O)	8.0 ± 2.2	8.3 ± 1.2	7.9 ± 1.8
PEEP level (cmH_2_O)	7.3 ± 1.0	7.0 ± 1.0	7.5 ± 1.1
Primary problems at admission to RCC (*n*)			
Pulmonary system	7 (41.2%)	9 (56.3%)	9 (52.9%)
Cardiovascular system	1 (5.9%)	2 (12.5%)	1 (5.9%)
Neurological system	7 (41.2%)	3 (18.6%)	3 (17.6%)
Gastrointestinal system	1 (5.9%)	1 (6.3%)	2 (11.8%)
Others	1 (5.9%)	1 (6.3%)	2 (11.8%)

IPPB: intermittent positive pressure breathing; PEEP: positive end-expiratory pressure; APACHE II: Acute Physiology and Chronic Health Evaluation II; CVA: cerebrovascular accident; DM: diabetes mellitus; ESRD: end stage renal disease; CHF: congestive heart failure; COPD: chronic obstructive pulmonary disease; CAD: coronary artery disease; RCC: respiratory care center.

All values are expressed as mean ± SD or number of patients (%).

**Table 2:** Comparison of weaning parameters and lung mechanics values between groups.

	IPPB (*n* = 17)	IPPB + PEEP (*n* = 16)	Control (*n* = 17)
Weaning profiles			
*V*_*t*_ (mL)			
Pre	240.4 ± 57.2	221.8 ± 157.4	293.9 ± 168.9
Post	292.5 ± 116.3^*∗*^	223.9 ± 110.3	243.9 ± 140.4^*∗*^
RR (bpm)			
Pre	25.4 ± 10.9	24.2 ± 11.7	23.6 ± 8.0
Post	22.8 ± 6.6	23.1 ± 9.4	25.2 ± 6.5
MV (L)			
Pre	6.6 ± 2.8	4.3 ± 1.4	6.6 ± 3.3
Post	6.1 ± 2.8	4.7 ± 2.9	5.8 ± 3.3
RSBI			
Pre	111.3 ± 49.5	161.8 ± 71.2	102.2 ± 62.4
Post	87.5 ± 25.4	137.4 ± 93.2	135.9 ± 82.2
MIP (cmH_2_O)			
Pre	33.8 ± 13.7	29.9 ± 15.0	38.0 ± 18.3
Post	36.9 ± 9.8	37.0 ± 16.5^*∗*^	40.6 ± 17.2
Lung mechanics			
Lung compliance (mL/cmH_2_O)			
Pre	48.2 ± 22.7	40.9 ± 22.4	34.4 ± 10.6
Post	55.4 ± 37.2	44.8 ± 24.8	33.9 ± 11.3
Airway resistance (cmH_2_O/L/s)			
Pre	7.6 ± 4.7	9.7 ± 5.2	9.7 ± 4.7
Post	7.9 ± 6.1	8.8 ± 3.9	9.3 ± 6.5

*V*_*t*_: tidal volume; RR: respiratory rate; MV: minute volume; RSBI: rapid shallow breathing index; MIP: maximal inspiratory pressure.

All values are expressed as mean ± SD. ^*∗*^Within-group comparison, pre versus post, *p* < 0.05.

**Table 3:** Comparison of arterial blood gas data between groups.

	IPPB (*n* = 17)	IPPB + PEEP (*n* = 16)	Control (*n* = 17)
Arterial blood gas (ABG)			
pH			
Pre	7.42 ± 0.12	7.47 ± 0.03	7.46 ± 0.05
Post	7.43 ± 0.04	7.42 ± 0.05^*∗*^	7.40 ± 0.06
PaCO_2_ (mmHg)			
Pre	40.4 ± 7.6	40.9 ± 5.8	38.1 ± 5.4
Post	44.5 ± 10.9	46.1 ± 13.5	40.3 ± 8.1
PaO_2_ (mmHg)			
Pre	98.1 ± 21.9	114.1 ± 30.0	111.0 ± 37.1
Post	122.1 ± 32.4^*∗*^	128.8 ± 49.3	106.7 ± 12.3
PaO_2_/FiO_2_			
Pre	275.6 ± 64.5	335.5 ± 98.6	327.6 ± 122.9
Post	381.2 ± 112.2^*∗*^	359.5 ± 124.7	287.4 ± 30.1

IPPB: intermittent positive pressure breathing; PEEP: positive end-expiratory pressure; ABG: arterial blood gas.

All values are expressed as mean ± SD. ^*∗*^Within-group comparison, pre versus post, *p* < 0.05.

**Table 4:** Comparison of hospitalization outcomes between groups.

	IPPB	IPPB + PEEP	Control
Weaning rate (%)	88.2% (15/17)	87.5% (14/16)	41.1% (7/17)^*∗*^
Ventilator days during RCC (days)	11.7 ± 3.7	15.8 ± 9.1	27.2 ± 16.1^*∗*^
Length of stay during RCC (days)	24.9 ± 10.7	23.6 ± 8.6	31.2 ± 13.1
Mortality rate (%)	0% (0/17)	0% (0/16)	5.9% (1/17)

RCC: respiratory care center.

All values are expressed as percentage (numbers) or mean ± SD.

^*∗*^Between-groups comparison, *p* < 0.05.

## References

[1] MacIntyre N. R., Epstein S. K., Carson S., Scheinhorn D., Christopher K., Muldoon S. (2005). Management of patients requiring prolonged mechanical ventilation: report of a NAMDRC Consensus Conference. *Chest*.

[2] Zilberberg M. D., Shorr A. F. (2008). Prolonged acute mechanical ventilation and hospital bed utilization in 2020 in the United States: implications for budgets, plant and personnel planning. *BMC Health Services Research*.

[3] Kao K.-C., Hu H.-C., Fu J.-Y. (2011). Renal replacement therapy in prolonged mechanical ventilation patients with renal failure in Taiwan. *Journal of Critical Care*.

[4] Wu Y.-K., Kao K.-C., Hsu K.-H., Hsieh M.-J., Tsai Y.-H. (2009). Predictors of successful weaning from prolonged mechanical ventilation in Taiwan. *Respiratory Medicine*.

[5] Carson S. S. (2006). Outcomes of prolonged mechanical ventilation. *Current Opinion in Critical Care*.

[6] Sackner M. A., Hirsch J., Epstein S. (1975). Effect of cuffed endotracheal tubes on tracheal mucous velocity. *Chest*.

[7] Hermans G., Agten A., Testelmans D., Decramer M., Gayan-Ramirez G. (2010). Increased duration of mechanical ventilation is associated with decreased diaphragmatic force: a prospective observational study. *Critical Care*.

[8] van Kaam A. H., Lachmann R. A., Herting E. (2004). Reducing atelectasis attenuates bacterial growth and translocation in experimental pneumonia. *American Journal of Respiratory and Critical Care Medicine*.

[9] Scheinhorn D. J., Hassenpflug M. S., Votto J. J. (2007). Ventilator-dependent survivors of catastrophic illness transferred to 23 long-term care hospitals for weaning from prolonged mechanical ventilation. *Chest*.

[10] Chen Y.-H., Lin H.-L., Hsiao H.-F. (2012). Effects of exercise training on pulmonary mechanics and functional status in patients with prolonged mechanical ventilation. *Respiratory Care*.

[11] Robert M., James K., Robert L. (2009). *Egan's Fundamentals of Respiratory Care*.

[12] Sinha R., Bergofsky E. H. (1972). Prolonged alteration of lung mechanics in kyphoscoliosis by positive pressure hyperinflation. *American Review of Respiratory Disease*.

[13] Dohna-Schwake C., Ragette R., Teschler H., Voit T., Mellies U. (2006). IPPB-assisted coughing in neuromuscular disorders. *Pediatric Pulmonology*.

[14] Andersen J. B., Jespersen W. (1980). Demonstration of intersegmental respiratory bronchioles in normal human lungs. *European Journal of Respiratory Diseases*.

[15] Guérin C., Vincent B., Petitjean T. (2010). The short-term effects of intermittent positive pressure breathing treatments on ventilation in patients with neuromuscular disease. *Respiratory Care*.

[16] Laffont I., Bensmail D., Lortat-Jacob S. (2008). Intermittent positive-pressure breathing effects in patients with high spinal cord injury. *Archives of Physical Medicine and Rehabilitation*.

[17] Sorensom H. M., Shelledy D. C. (2003). AARC clinical practice guidelines: Intermittent positive pressure breathing—2003 revision and update. *Respiratory Care*.

[18] Romanini W., Muller A. P., de Carvalho K. A. T. (2007). The effects of intermittent positive pressure and incentive spirometry in the postoperative of myocardial revascularization. *Arquivos Brasileiros de Cardiologia*.

[19] Pilbeam S. P., Cairo J. M. (2006). *Mechanical Ventilation: Physiological and Clinical Applications*.

[20] Unroe M., Kahn J. M., Carson S. S. (2010). One-year trajectories of care and resource utilization for recipients of prolonged mechanical ventilation. A cohort study. *Annals of Internal Medicine*.

[21] Truong A. D., Fan E., Brower R. G., Needham D. M. (2009). Bench-to-bedside review: mobilizing patients in the intensive care unit—from pathophysiology to clinical trials. *Critical Care*.

[22] Brower R. G. (2009). Consequences of bed rest. *Critical Care Medicine*.

[23] Örman J., Westerdahl E. (2010). Chest physiotherapy with positive expiratory pressure breathing after abdominal and thoracic surgery: a systematic review. *Acta Anaesthesiologica Scandinavica*.

[24] Johnson P. H., Cowley A. J., Kinnear W. J. (1996). Evaluation of the threshold trainer for inspiratory muscle endurance training: comparison with the weighted plunger method. *European Respiratory Journal*.

[25] Chen Y. C., Wu L. F., Mu P. F. (2009). Chest vibration nursing intervention to improve expectoration of airway secretions and prevent lung collapse in ventilated ICU patients: a randomized controlled trial. *Journal of the Chinese Medical Association*.

[26] Celli B. R., Rodriguez K. S., Snider G. L. (1984). A controlled trial of intermittent positive pressure breathing, incentive spirometry, and deep breathing exercises in preventing pulmonary complications after abdominal surgery. *American Review of Respiratory Disease*.

